# Draft Genome Sequences of *Synechococcus* sp. strains CCAP1479/9, CCAP1479/10, CCAP1479/13, CCY0621, and CCY9618: Five Freshwater *Syn/Pro* Clade Picocyanobacteria

**DOI:** 10.7150/jgen.81013

**Published:** 2023-04-25

**Authors:** Elliot Druce, Michele Grego, Henk Bolhuis, Penny J. Johnes, Patricia Sánchez-Baracaldo

**Affiliations:** 1 School of Geographical Sciences, Faculty of Science, University of Bristol, Bristol, BS8 1SS, United Kingdom.; 2 CNRS and Sorbonne Université, FR 2424, Roscoff Culture Collection, Station Biologique de Roscoff (SBR), Roscoff, France; 3 Department of Marine Microbiology and Biogeochemistry, Royal Netherlands Institute for Sea Research, Den Hoorn, the Netherlands.

**Keywords:** Freshwater, Picocyanobacteria, *Synechococcus*, Genome, *Synechococcus sp. CCAP1479/9*, * Synechococcus* sp. CCAP1479/10, *Synechococcus sp. CCAP1479/13*, * Synechococcus* sp. CCY0621, *Synechococcus* sp. CCY9618

## Abstract

Picocyanobacteria are essential primary producers in freshwaters yet little is known about their genomic diversity and ecological niches. We report here five draft genomes of freshwater picocyanobacteria: *Synechococcus* sp. CCAP1479/9, *Synechococcus* sp. CCAP1479/10, and *Synechococcus* sp. CCAP1479/13 isolated from Lake Windermere in the Lake District, UK; and *Synechococcus* sp. CCY0621 and *Synechococcus* sp. CCY9618 isolated from lakes in The Netherlands. Phylogenetic analysis reveals all five strains belonging to sub-cluster 5.2 of the *Synechococcus* and *Prochlorococcus* clade of Cyanobacteria. These five strains are divergent from *Synechococcus elongatus*, an often-used model for freshwater *Synechococcus*. Functional annotation revealed significant differences in the number of genes involved in the transport and metabolism of several macro-molecules between freshwater picocyanobacteria from sub-cluster 5.2 and *Synechococcus elongatus*, including amino acids, lipids, and carbohydrates. Comparative genomic analysis identified further differences in the presence of photosynthesis-associated proteins while gene neighbourhood comparisons revealed alternative structures of the nitrate assimilation operon *nirA*.

## Introduction

Picocyanobacteria play a key role in aquatic ecosystems, contributing a significant proportion of total primary production in both marine and fresh waters 1-3. These unicellular cyanobacteria, sized between 0.5 and 2 μm, are distributed globally, from temperate and tropical open oceans to alpine lakes and eutrophic reservoirs 4-6. Freshwater picocyanobacteria are predominantly *Synechococcus* strains which can dominate the picophytoplankton component (1 - 99% 7) and total biomass (10 - 70% 8) depending on trophic status and depth 9,10. Other taxonomic names associated with freshwater picocyanobacterial strains are *Cyanobium* spp. 11 and *Vulcanococcus* spp. 12.

The availability of sequenced freshwater picocyanobacteria genomes has lagged behind that of marine picocyanobacteria (*Prochlorococcus* and *Synechococcus*) 13. This has limited genomic approaches to understand freshwater picocyanobacteria with regards to ecology and evolution - a hot topic in both freshwater and marine environments 14-18. A further limitation is the divergence seen among freshwater *Synechococcus* clades. Though *Synechococcus elongatus* cells are larger than those of the *Syn/Pro* clade *Synechococcus* 19,20, and do not fall under the 'pico-' threshold, they are often used as models for freshwater picocyanobacteria 21-25. However, the emergence of the *Synechococcus elongatus* strains as a deep branching sister group to the monophyletic *Syn/Pro* clade suggests *Synechococcus elongatus* provides an unrepresentative model of freshwater picocyanobacteria and freshwater *Synechococcus* 26. Freshwater strains of the *Syn/Pro* clade have a wider geographic distribution than *Synechococcus elongatus* and may have a greater ecological influence 27, yet their molecular capabilities are poorly understood in comparison to *Synechococcus elongatus*. Here, we have sequenced draft genomes of five new picocyanobacteria to increase genomic representation of the freshwater strains in the *Syn/Pro* clade. Three were isolated from Lake Windermere in the UK: *Synechococcus* sp. CCAP1479/9, *Synechococcus* sp. CCAP1479/10, and *Synechococcus* sp. CCAP1479/13. The remaining two were isolated from ponds in the Netherlands: *Synechococcus* sp. CCY0621 (Leiden) and *Synechococcus* sp. CCY9618 (Vinkeveen).

## Materials and Methods

Three *Synechococcus* strains were obtained from the Culture Collection of Algae and Protozoa: *Synechococcus* sp. CCAP1479/9, *Synechococcus* sp. CCAP1479/10, and *Synechococcus* sp. CCAP1479/13, all isolated from Lake Windermere, UK. Two *Synechococcus* strains were obtained from the Culture Collection Yerseke: *Synechococcus* sp. CCY0621 and *Synechococcus* sp. CCY9618, isolated from ponds in The Netherlands (Leiden and Vinkeveen respectively) (Supplementary Figure S1). All strains were grown in BG-11 medium 28 at 20 °C with 10-20 µmol m^-2^ s^-1^ of white light under a 16 h: 8 h light:dark cycle.

Aliquots of 1.8 mL of each mono-phototrophic culture were harvested to extract genomic DNA using DNeasy UltraClean Microbial Kits (Qiagen, Germany) according to the manufacturer's instructions. Once purified, genomic DNA was stored at -80 °C in 10 mM Tris buffer at pH 8. DNA concentration and quality was measured using a NanoDrop 2000 spectrophotometer (Thermo Scientific, USA) and a Qubit 2.0 Fluorometer (Thermo Scientific, USA).

Whole genome library preparation and sequencing was carried out by the University of Bristol Genomics Facility, UK. DNA libraries were prepared for each strain using Truseq Nano LT Kit (Illumina, USA) and sequenced using Illumina NextSeq 500/550 Mid Output Kit v2 (300 cycles) (Illumina, USA) to generate paired-end reads (2 x 150 bps). Raw reads were trimmed using Trimmomatic v0.39 29 with parameters Leading: 20, Trailing: 20, SlidingWindow: 4:20, MinLen: 20, and assembled de novo using SPAdes v3.14.1 30 with k-mers of 67, 77, 87, 97 and a coverage cutoff of 20 in --careful mode. A BLAST database was generated at the amino acid sequence level for each assembly and searched against a collection of 1,054 core cyanobacterial genes (CCGs) 31,32. Bandage v0.8.1 33 was used to visualise strain assemblies and separate out cyanobacterial sequences based on contiguous CCG-containing nodes as demonstrated in previous assemblies 32. Contigs which did not contain cyanobacterial genes were discarded, in addition to short (<200 bp) contigs. The assembled genomes had overall coverages ranging from 552x to 939x (Table 1) and structurally annotated with GeneMark.hmm-2 v1.05 34, Prodigal v2.6.3 35, INFERNAL v1.1.2 36, and tRNAscan-SE v2.0.5 37. Genome completeness was estimated by identifying cyanobacteria-specific single-copy orthologous genes using BUSCO v3.0.2 38. The draft genomes were submitted to JGI IMG/ER 39 (GOLD Analysis Project IDs: Ga0436386, Ga0436387, Ga0436388, Ga0436389, and Ga0436390). The five draft genomes were deposited to the DDBJ/Genbank/ENA repositories with accession numbers JAFKRG000000000 (CCY9618), JAFKRH000000000 (CCY0621), JAFKRI000000000 (CCAP1479/13), JAFKRJ000000000 (CCAP1479/10), and JAFKRK000000000 (CCAP1479/9).

Functional annotation was determined through the eggNOG web server 40. Two-tailed t-tests were applied to carry out statistical analysis on total COG numbers and COGs normalised as a proportion of total genome. JGI IMG/ER was used to carry out KEGG 41 comparative genomic analysis for photosynthesis and nitrate metabolism pathways between *Synechococcus elongatus* (*Synechococcus elongatus* PCC 7942, *Synechococcus elongatus* UTEX 2973, *Synechococcus elongatus* PCC 6301, *Synechococcus elongatus* FACHB-242,* Synechococcus elongatus* FACHB-1061) and the sequenced *Synechococcus* strains.

The evolutionary relationships of the newly sequenced strains with a selection of cyanobacterial taxa sampling a broad range of morphologies, lifestyles, and metabolisms, were estimated through phylogenetic analysis. Our dataset included 373 cyanobacteria genomes and ortholog sequences from 143 protein-coding genes, based on previously published studies 42-44. We performed BLAST searches with these ortholog sequences against the 373 genomes using blastp v2.11.0+ 45 with an e-value threshold of 10^-5^, retaining the hit with the highest score and extracting the corresponding protein sequences. The resulting sequences were aligned using MAFFT v7.511 46 with the -localpair -maxiterate 1000 parameters. Maximum-likelihood gene trees were constructed using IQ-TREE 2.2.0 47, implementing the *LG* protein evolution model and the -fast option. These gene trees were used to identify the clusters of sequences that were most closely associated with the BLAST query sequences - these clusters were assumed to be 'true' orthologs. These true orthologs were re-aligned with MAFFT (same parameters as above) and inspected with mis-aligned columns and alignment positions with a gap content higher than 80% removed from each alignment. The best evolutionary model for each gene was determined by using IQ-TREE with the -m MF option 48, selecting the model with the lowest BIC score. A maximum-likelihood partitioned phylogenetic analysis was performed using IQ-TREE 49. Using the previously determined evolutionary models, partitioned analysis was carried out with IQ-TREE using -p and -B 1000 parameters with each gene assigned to its own partition. The -p option constrains all partitions to the same topology and branch length but allows each partition to have a different overall evolutionary rate, while -B 1000 produces ultrafast bootstrap support values 50. This analysis was carried out twice with the two resulting trees compared to confirm no significant differences between them.

## Results and Discussion

The newly sequenced picocyanobacteria genomes consist of 88 to 133 contigs (average of 112) and range in size from 2.9 Mbps to 3.3 Mbps (average of 3.2 Mbps), significantly larger than *Synechococcus elongatus* strains (p < .001, n = 5). *Synechococcus* sp. CCY9618 has the smallest genome and is composed of the largest number of contigs with an N50 value of 94,487 (Table 1). *Synechococcus* sp. CCAP1479/10 has the largest genome, while *Synechococcus* sp. CCAP1479/9 contains the fewest contigs (88) and the largest N50 (207,208). Genome coverage is high among the assemblies (552x - 939x) with genome completeness estimated at 98.2 - 98.7%. It should be noted that these genomes have not been completely closed yet a high genome completeness suggests that the 'missing' part of the genome is limited.

**Figure 1:**
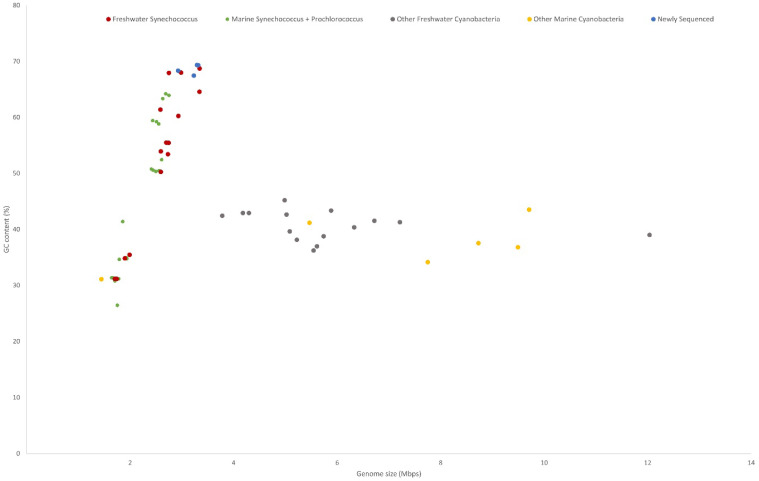
GC content and genome size of cyanobacteria characterised into habitat and phylogeny. Freshwater Synechococcus include picocyanobacteria found in the Syn/Pro clade and Synechococcus elongatus strains. The newly sequenced strains are clustered with the freshwater Synechococcus.

All five genomes contain high GC contents ranging from 67.45 - 69.36% (Table 1). This is consistent with previously sequenced freshwater picocyanobacteria and *Synechococcus elongatus*, regularly featuring a high (>60%) GC content 13,18. Compared to marine *Syn/Pro* strains, freshwater *Synechococcus* have significantly larger genome sizes (p < .001, n = 20; primarily due to genomic streamlining of *Prochlorococcus* spp. 51) and higher GC content (p < .001, n = 20) (Figure 1). Meanwhile, the trend of increasing GC content with increasing genome size present in freshwater and marine *Synechococcus* is not found in larger cyanobacteria (cell size greater than 2 µm). Higher genomic GC contents have been linked with increased horizonal gene transfer and protection against DNA damage through higher resilience against UV irradiation, contributing to picocyanobacterial genomic plasticity and environmental adaptability 52,53. Conversely, lower GC contents in marine picocyanobacteria may indicate selection in N limited environments due to the reduced N requirement for AT pairs 54.

**Figure 2:**
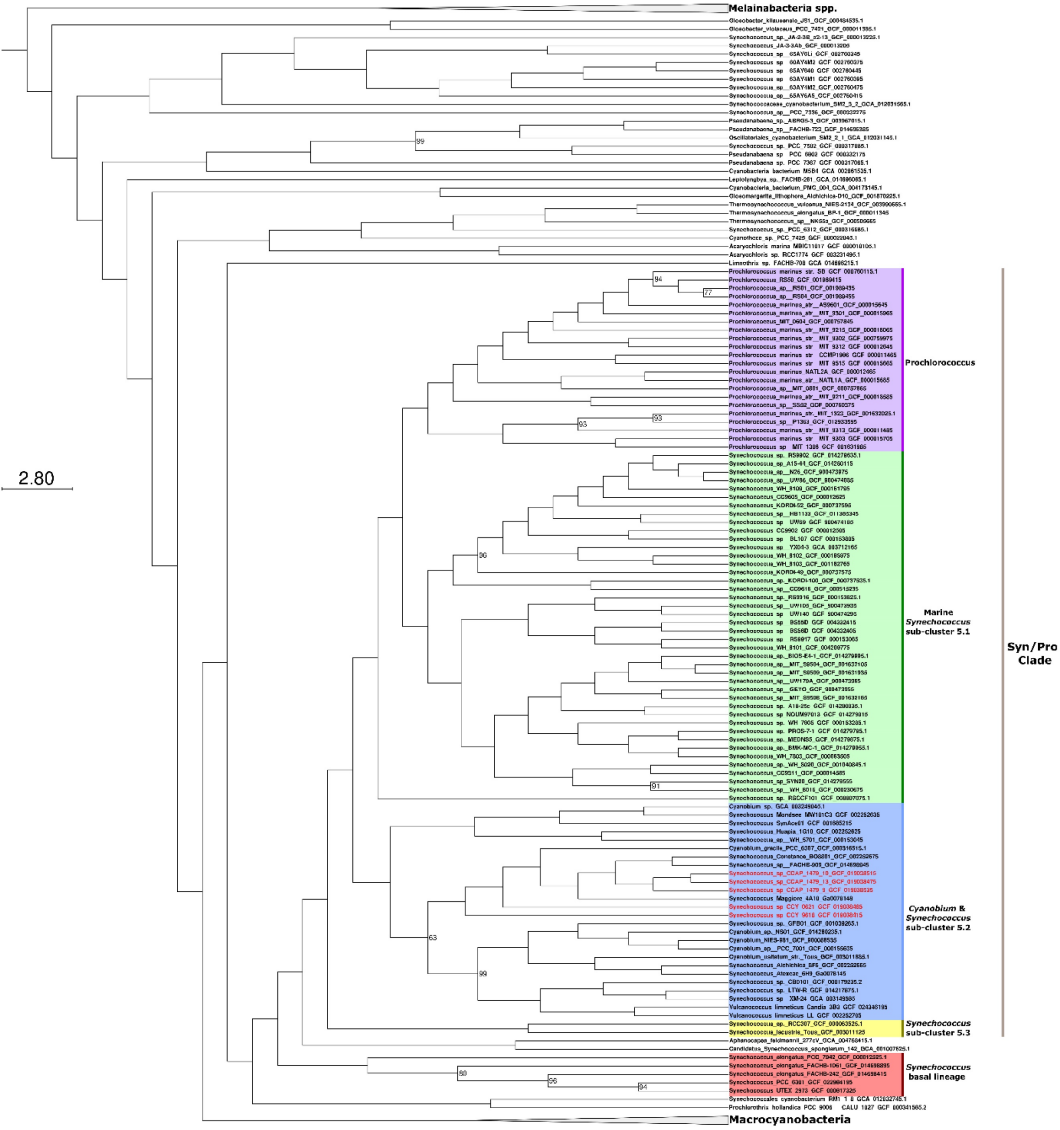
Maximum likelihood phylogeny showing the relationship of Synechococcus sp. CCAP 1479/9, Synechococcus sp. CCAP 1479/10, Synechococcus sp. CCAP 1479/13, Synechococcus sp. CCY 0621, and Synechococcus sp. CCY 9618 within the Syn/Pro clade. Newly sequenced picocyanobacteria are highlighted in red. The tree was constructed from 373 cyanobacteria and 145 orthologous proteins. Bootstrap values less than 100 are displayed at branching nodes while blank nodes have a support of 100. The tree is rooted using Melainabacteria spp. as an outgroup. The scale bar represents an average of 2.8 substitutions per site. An expanded tree is shown in Supplementary Information Figure S2.

Phylogenomic analyses were carried out to identify the closest relatives of the newly sequenced freshwater picocyanobacteria. All five strains belong to the *Cyanobium* and *Synechococcus* freshwater sub-cluster 5.2 of the *Syn/Pro* clade (Figure 2). *Synechococcus* sp. CCAP1479/10, *Synechococcus* sp. CCAP1479/13, and *Synechococcus* sp. CCAP1479/13 form a monophyletic clade, with *Synechococcus* sp. BO8801 (Lake Constance, Germany) and *Synechococcus* sp. FACHB-909 (Baohu Lake, China) the closest related strains (a sister group to these three newly sequenced picocyanobacteria). *Synechococcus* sp. CCY0621 and *Synechococcus* sp. CCY9618 are more distantly related and appear as outgroups to the CCAP newly sequenced strains. In contrast, *Synechococcus elongatus* strains are a sister group of the *Syn/Pro.*

Freshwater picocyanobacteria from the *Syn/Pro* clade are derived taxa that specialised in a planktonic habitat. The newly sequenced genomes were functionally annotated with eggNOG and KEGG, in addition to five *Synechococcus elongatus* genomes (*Synechococcus elongatus* PCC 7942, *Synechococcus elongatus* UTEX 2973,* Synechococcus elongatus* PCC 6301, *Synechococcus elongatus* FACHB-242, and *Synechococcus elongatus* FACHB-1061). This enabled insights into the genomic capabilities of the scarcely researched freshwater sub-cluster 5.2 of the *Syn/Pro* clade compared to the *Synechococcus elongatus* basal lineage.

Of the 19 functional COG categories identified, 11 categories differed significantly between our sequenced genomes and *Synechococcus elongatus* strains, in terms of total gene number and genes as a percentage of the total genome (Table 2, Supplementary Table S1). Five of these categories were found to be significantly increased in our sequenced genomes (V, M, G, E, I), while three were significantly decreased (J, N, F). The total number of genes associated with three categories (O, C, H) were significantly greater in our sequenced genomes (p < .001, n = 5), though as a proportion of their genome were significantly greater in *Synechococcus elongatus* strains (p = .006, p = .007, p < .001 respectively, all n = 5). Additionally, KEGG analysis revealed 1,425 KO terms within at least one of the sub-cluster 5.2 freshwater picocyanobacteria of which 183 terms were not identified in *Synechococcus elongatus* strains. Meanwhile, 162 KO terms are found in *Synechococcus elongatus* but absent from our newly sequenced strains (Supplementary Table S2).

Our sequenced sub-cluster 5.2 strains encode significantly more genes involved in carbohydrate (G), amino acid (E), and lipid (I) transport and metabolism than *Synechococcus elongatus* strains (p < .001, n = 5). Conversely, *Synechococcus elongatus* strains encode significantly more nucleotide transport and metabolism genes (F; p < .001, n = 5). As the *Synechococcus elongatus* genome size is smaller than that of our sub-cluster 5.2 freshwater strains, it may be expected to encode a reduced number of nucleotide-associated genes though this is not found. These genomic differences may be caused by the different environmental niches these two clades inhabit. Fresh waters are spatially diverse and exhibit a greater amount of nutrient heterogeneity than ocean environments 55. Multiple other factors contribute to freshwater habitat niches, including light availability, temperature, water retention time, and composition of the surrounding microbial community 56. However, while sub-cluster 5.2 and *Synechococcus elongatus* strains have been isolated from geographically distant locations, they occupy the same position in the water column (limnetic zone based on presence of phycocyanin 13) and are more dominant in temperate waters. Increased genomic sequencing of taxa from sub-cluster 5.2 will aid in understanding freshwater picocyanobacteria ecology and the evolutionary context of these divergent lineages.

Further differences have been identified in the number of genes responsible for information storage and cellular processes between our sequenced strains and *Synechococcus elongatus* strains. Genes encoding defence mechanisms (V) and cell wall biogenesis-related (M) proteins are significantly increased in our newly sequenced strains (p < .05, n = 5). Meanwhile, *Synechococcus elongatus* strains have significantly higher numbers of genes involved in translation (J) and cell motility (N) (p < .001, n = 5). Research on cyanobacterial chemo- and photo-taxis has focused on *Synechocystis* spp. which exhibit a 'gliding' form of motility utilising a type IV pilus system 57. Motility among marine *Synechococcus* spp. is achieved through multiple mechanisms, the most common through S-layer rotation 58,59, while recent findings have identified phototactic behaviour in *Synechococcus elongatus* 60. However, the motility of sub-cluster 5.2 is yet to be determined. These differences in core cellular control may represent subtle changes in clade behaviour. As *Synechococcus elongatus* PCC 7942 is traditionally used as a model for freshwater *Synechococcus*, the variations in the genome may distort expectations of the *Syn/Pro* clade.

A comparison of the photosynthesis pathway between the newly sequenced picocyanobacteria and *Synechococcus elongatus* reveals a number of differences. Among core Photosystem II (PSII) components, the gene for the D2 protein (*psbD*) is surprisingly absent from the newly sequenced strains (in addition to two recently sequenced *Synechococcus elongatus*) (Table 3). The D2 protein forms part of the PSII reaction core alongside D1 (encoded by *psbA*) and is essential in binding the necessary redox-active cofactors for electron transfer 61. The presence of *psbD* in other sub-cluster 5.2 strains is likewise unclear - absent from *Synechococcus* sp. BO8801 yet found in *Synechococcus* sp. 1G10 and *Cyanobium gracile* PCC 6307 (data not shown). However, the absence of *psbD* from our sequenced picocyanobacteria may be a result of the unclosed nature of the genome. *psbC* is found clustered with *psbD* in other cyanobacteria (e.g., *Synechococcus elongatus* PCC 7942 and *Synechocystis* sp. PCC 6803), though the contig encoding *psbC* in our sequenced *Synechococcus* spp. is truncated upstream (where the *psbD* locus is usually found). Other genes encoding photosynthesis electron transport proteins that are absent from our newly sequenced sub-cluster 5.2 strains include p*etL* encoding the cytochrome b6f complex subunit 6, and *petE* encoding plastocyanin, responsible for transferring electrons from cytochrome b6f to Photosystem I (PSI). Cytochrome b6f is an intermediate in the transport of electrons from PSII to PSI, however the role of PetL in the complex is unclear. A function linked to stability of the dimeric state of the cytochrome b6f complex has been suggested while the non-essential nature of PetL in cyanobacteria has been demonstrated 62,63. Accepting electrons from cytochrome b6f, copper-containing plastocyanin is another essential component of the photosynthesis electron transport chain. However, most cyanobacteria also contain Fe-containing cytochrome c6 (encoded by *petJ*). Expression of these two electron carriers is regulated by copper availability, a response to Fe-limitation 64. The absence of plastocyanin in sub-cluster 5.2 strains appears to reduce adaptability in low-Fe environments, though heterocyst-forming cyanobacteria have been shown to preferentially utilise cytochrome c6 for electron transport, even in the presence of copper 65. While the deletion of *psbD* is likely an artefact and must be resolved by the generation of closed freshwater picocyanobacteria genomes, further research to investigate the impact of the putative *petL* and *petE* gene deletions is necessary to elucidate this key physiological process in freshwater picocyanobacteria.

In addition to core photosynthetic electron transport apparatus, the copy number and composition of antennae proteins comprising the light-harvesting phycobilisome (PBS) displays subtle differences (Table 3). *Synechococcus elongatus* strains encode two copies of *apcD*, encoding a key component of the allophycocyanin (AP) central core of PBS, though our newly sequenced sub-cluster 5.2 picocyanobacteria encode solely *apcD1*. The role of ApcD has been shown to slightly vary between *Synechococcus elongatus* PCC 7942 and another cyanobacterial model organism - *Synechocystis* sp. PCC 6803. ApcD is vital for efficient energy transfer from the PBS to PSI in *Synechococcus elongatus* PCC 7942 while the lack of ApcD has no impact on PSI energy transfer in *Synechocystis* sp. PCC 6803, instead inhibiting state transitions in response to unbalanced light conditions 66. Furthermore, multiple copies of *apcD* have been linked to photoacclimation to far-red light (700 - 750 nm), aiding absorbance of a greater diversity of wavelengths 67. This may suggest a wider range of utilisable wavelengths for *Synechococcus elongatus* strains, resulting in community shifts in heavily shaded areas.

There are more significant variations in the encoding of phycobiliprotein-rods which radiate out from the PBS core. There are differences in the copy number of phycocyanin (PC) subunits *cpcA* and *cpcB* with *Synechococcus* sp. CCY9618 encoding only *cpcB.* Other newly sequenced genomes encode both subunits with *cpcB* at an increased copy number compared to *Synechococcus elongatus* strains (Table 3). Interestingly, *cpcC* is absent from our sub-cluster 5.2 strains. This encodes the LR33 PC-associated linker polypeptide, responsible for stabilising rod substructures 68. Meanwhile, the same strains encode an additional copy of *cpcG* (encoding a linker protein required for rod attachment to the AP core), with the two copies having distinct roles in PSII (*cpcG1*) and PSI (*cpcG2*) in *Synechocystis* sp. PCC 6803^69^. The absence of *cpcG2* in *Synechococcus elongatus* strains suggests further differences in photosynthetic machinery between the two groups. Furthermore, while phycoerythrin (PE) is known to be absent in *Synechococcus elongatus* strains, it has been observed in other sequenced sub-cluster 5.2 freshwater picocyanobacteria 13. However, the strains sequenced in this study are absent of *cpeAB* indicating PBS rods of PC only. Though lacking PE subunits, freshwater *Synechococcus* encode various PE-associated proteins. Our sequenced *Synechococcus* encode two copies of *cpeC*, a PE-associated rod linker protein, while *Synechococcus elongatus* encode *cpeS*, an S-type lyase essential for mature PE generation 70,71. It is unclear if these genes are expressed, and the function they provide for *Synechococcus* lacking PE.

**Figure 3:**
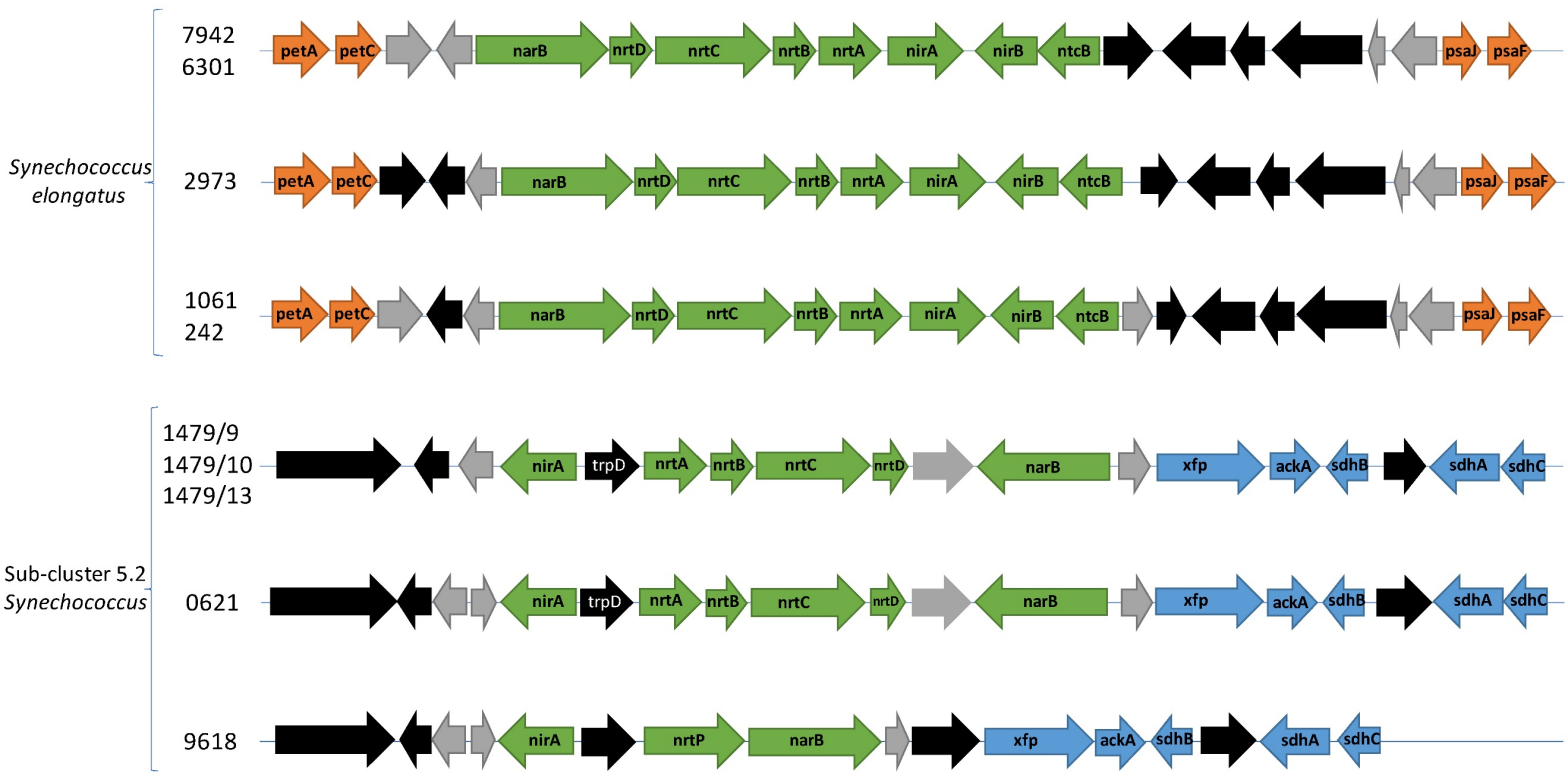
Gene neighbourhood of the narB-nrtABCD-nirA operon for nitrate assimilation. Green arrows are genes involved with nitrate assimilation. Orange arrows are genes involved with photosynthesis. Blue arrows are genes involved with carbon metabolism. Black arrows are other annotated genes while grey arrows indicate hypothetical genes. petA: apocytochrome f (K02634). petC: cytochrome b6f complex iron-sulphur subunit (K02636), psaJ: photosystem I subunit 9 (K02697), psaF: Photosystem I subunit 3 K02694), xfp: xylulose-5-phosphate/fructose-6-phosphate phosphoketolase (K01621), ackA: acetate kinase (K00925), sdhB: succinate dehydrogenase/fumarate reductase iron-sulphur subunit (K00240), sdhA: succinate dehydrogenase/fumarate reductase flavoprotein subunit (K00239), sdhC: succinate dehydrogenase/fumarate reductase cytochrome b subunit (K00241).

The most abundant N source in fresh water is nitrate 72, a nutrient which cyanobacteria can access via the *narB-nrtABCD-nirA* operon. This operon encodes the necessary proteins for nitrate assimilation, yet the gene neighbourhood of this operon differs between sub-cluster 5.2 freshwater picocyanobacteria and *Synechococcus elongatus*. This operon consists of a nitrate/nitrite bi-specific ABC-type transporter (*nrtABCD*), nitrate reductase (*narB*), and nitrite reductase (*nirA*). Among our newly sequenced strains (apart from *Synechococcus* sp. CCY 9618), *nirA* and *narB* are transcribed in the opposite direction to *nrtABCD* whereas *Synechococcus elongatus* encodes the six core genes continuously (Figure 3). Furthermore, there are unrelated genes flanking *nrtABCD* - anthranilate phosphoribosyltransferase and a hypothetical gene. Contiguous operons are known for rapid gene expression for all proteins of a specific cellular process, however the unassociated genes and two-way transcription may suggest sub-cluster 5.2 freshwater picocyanobacteria respond slower to nitrate inducement, though bidirectional promoters may be involved.

Additional genes involved with nitrite assimilation are found in *Synechococcus elongatus* strains but absent from our sequenced strains. These include *nirB*, required for maximal nitrite reductase activity, and *ntcB*, a transcription factor involved in nitrite-induced gene activation 73,74. Though nitrate is the most abundant traditional N source, it is also the most energetically costly, requiring eight electrons to reduce fully to ammonium (nitrate > nitrite > ammonium). Increasing the preference for nitrite over nitrate can reduce this demand which may result in substantial energy savings. *Synechococcus* sp. CCY9618 encodes a homologous transporter previously only identified in marine picocyanobacteria, *nrtP*, which preferentially takes up nitrate over nitrite 75. The differences between sub-cluster 5.2 freshwater picocyanobacteria and *Synechococcus elongatus* may indicate differing preferences for nutrient growth, influencing the composition of the *Synechococcus* community.

The newly sequenced five freshwater picocyanobacteria expand the number of genomes available for sub-cluster 5.2 of the *Syn/Pro* clade. The number of genomic capabilities for metabolism and cellular processes vary significantly between these strains and *Synechococcus elongatus* strains. These findings contribute to a better understanding not only of the ecology, but the evolutionary relationships of freshwater *Synechococcus* and re-evaluates the conclusions that can be drawn from the model organism *Synechococcus elongatus*.

## Supplementary Material

Supplementary tables.

Supplementary figures 1, 3-7.

Supplementary figure 2.

## Figures and Tables

**Table 1:** Genomic features of the sequenced freshwater picocyanobacteria

	*Synechococcus* sp. CCAP1479/9	*Synechococcus* sp. CCAP1479/10	*Synechococcus* sp. CCAP1479/13	*Synechococcus* sp. CCY0621	*Synechococcus* sp. CCY9618
Genome size (bp)	3,288,920	3,313,705	3,299,582	3,230,971	2,927,161
Contigs	88	108	132	101	133
N50 (bp)	207,208	151,487	78,719	105,719	94,487
Genome coverage	825X	939X	552X	818X	865X
DNA coding (%)	91.98	91.99	91.92	90.96	90.5
DNA G+C (%)	69.36	69.33	69.32	67.45	68.34
Total genes	3,423	3,502	3,507	3,471	3,165
Protein encoding genes	3,364	3,441	3,446	3,407	3,109
Completeness (%)	98.4	98.7	98.2	98.6	98.6
Average Nucleotide Identity to *Synechococcus elongatus* PCC 7942	73.5808	73.4985	73.5014	73.3739	73.3276

**Table 2:** Number of eggNOG classifications of proteins encoded by the five sequenced sub-cluster 5.2 *Synechococcus* genomes and five selected *Synechococcus elongatus* strains*.* Percentage of genes as proportion of the genome is provided in brackets. J: Translation, ribosomal structure and biogenesis; K: Transcription; L: Replication, recombination and repair; B: Chromatin structure and dynamics; D: Cell cycle control, cell division, chromosome partitioning; V: Defence mechanisms; T: Signal transduction mechanisms; M: Cell wall/membrane/envelope biogenesis; N: Cell motility; U: Intracellular trafficking, secretion, and vesicular transport; O: Posttranslational modification, protein turnover, chaperones; C: Energy production and conversion; G: Carbohydrate transport and metabolism; E: Amino acid transport and metabolism; F: Nucleotide transport and metabolism; H: Coenzyme transport and metabolism; I: Lipid transport and metabolism; P: Inorganic ion transport and metabolism; Q: Secondary metabolites biosynthesis, transport and catabolism; S: Function unknown.

COG	*Synechococcus* sp. CCAP1479/9	*Synechococcus* sp. CCAP1479/10	*Synechococcus* sp. CCAP1479/13	*Synechococcus* sp. CCY0621	*Synechococcus* sp. CCY9618	*Synechococcus elongatus* PCC 7942	*Synechococcus elongatus* UTEX 2973	*Synechococcus elongatus* PCC 6301	*Synechococcus elongatus* FACHB-242	*Synechococcus elongatus* FACHB-1061
J	163 (4.7)	161 (4.7)	161 (4.7)	161 (4.7)	160 (5.1)	163 (6.1)	166 (6.1)	165 (6.5)	167 (6)	167 (6)
K	161 (4.8)	155 (4.5)	154 (4.5)	138 (4.1)	116 (3.7)	114 (4.3)	115 (4.2)	110 (4.4)	114 (4.1)	114 (4.1)
L	116 (3.4)	121 (3.5)	122 (3.5)	127 (3.7)	146 (4.7)	108 (4.1)	116 (4.3)	112 (4.4)	114 (4.1)	114 (4.1)
B	2 (0.1)	2 (0.1)	2 (0.1)	2 (0.1)	2 (0.1)	2 (0.1)	2 (0.1)	2 (0.1)	2 (0.1)	2 (0.1)
D	35 (1)	42 (1.2)	42 (1.2)	37 (1.1)	28 (0.9)	27 (1)	27 (1)	25 (1)	27 (1)	27 (1)
V	41 (1.2)	43 (1.2)	43 (1.2)	51 (1.5)	47 (1.5)	31 (1.2)	31 (1.1)	31 (1.2)	31 (1.1)	31 (1.1)
T	92 (2.7)	100 (2.9)	98 (2.8)	89 (2.6)	62 (2)	85 (3.2)	117 (4.3)	109 (4.3)	117 (4.2)	117 (4.2)
M	199 (5.9)	201 (5.8)	203 (5.9)	204 (6)	186 (6)	134 (5)	148 (5.4)	145 (5.7)	148 (5.3)	148 (5.3)
N	19 (0.6)	21 (0.6)	20 (0.6)	17 (0.5)	18 (0.6)	26 (1)	26 (1)	26 (1)	26 (0.9)	25 (0.9)
U	71 (2.1)	73 (2.1)	75 (2.2)	71 (2.1)	62 (2)	34 (1.3)	55 (2)	53 (2.1)	55 (2)	54 (2)
O	111 (3.3)	114 (3.3)	115 (3.3)	114 (3.3)	108 (3.5)	94 (3.5)	99 (3.6)	101 (4)	100 (3.6)	100 (3.6)
C	220 (6.5)	229 (6.7)	230 (6.7)	220 (6.5)	224 (7.2)	199 (7.5)	199 (7.3)	202 (8)	198 (7.2)	199 (7.2)
G	112 (3.3)	111 (3.2)	113 (3.3)	104 (3.1)	103 (3.3)	71 (2.7)	82 (3)	80 (3.2)	82 (3)	82 (3)
E	181 (5.4)	184 (5.3)	181 (5.3)	174 (5.1)	163 (5.2)	138 (5.2)	137 (5)	132 (5.2)	138 (5)	138 (5)
F	86 (2.6)	89 (2.6)	89 (2.6)	88 (2.6)	83 (2.7)	98 (3.7)	97 (3.6)	96 (3.8)	98 (3.5)	98 (3.5)
H	186 (5.5)	183 (5.3)	185 (5.4)	185 (5.4)	181 (5.8)	167 (6.3)	171 (6.3)	171 (6.8)	173 (6.3)	173 (6.3)
I	79 (2.3)	80 (2.3)	79 (2.3)	88 (2.6)	70 (2.3)	54 (2)	54 (2)	51 (2)	54 (2)	54 (2)
P	142 (4.2)	151 (4.4)	151 (4.4)	162 (4.8)	122 (3.9)	152 (5.7)	163 (6)	157 (6.2)	163 (5.9)	164 (5.9)
Q	46 (1.4)	45 (1.3)	45 (1.3)	46 (1.4)	39 (1.3)	27 (1)	44 (1.6)	42 (1.7)	44 (1.6)	44 (1.6)
S	669 (19.9)	711 (20.7)	710 (20.6)	704 (20.7)	629 (20.2)	572 (21.5)	737 (27.1)	715 (28.3)	745 (26.9)	742 (36.8)

**Table 3:** Genes encoding photosynthesis machinery and antennae proteins found in the five sequenced sub-cluster 5.2 *Synechococcus* genomes and five selected *Synechococcus elongatus* strains. Genes were identified through KEGG annotation. Copy number is indicated by the number of '+' symbols. Absence of the gene indicated by '-'.

		*Synechococcus* sp.					*Synechococcus elongatus*				
Kegg Orthology (KO)	Gene Product	CCAP 1479/9	CCAP 1479/10	CCAP 1479/13	CCY 0621	CCY 9618	PCC 7942	UTEX 2973	PCC 6301	FACHB-242	FACHB-1061
Photosynthesis											
PSII											
K02703	PsbA	++	+++	+++	+++	++	+++	+++	+++	+++	++
K02706	PsbD	-	-	-	-	-	++	++	++	-	-
K02705	PsbC	+	+	+	+	+	+	+	+	+	+
K02704	PsbB	+	+	+	+	+	+	+	+	+	+
K02707	PsbE	+	+	+	+	+	+	+	+	+	+
K02708	PsbF	+	+	+	+	+	+	+	+	+	+
K02713	PsbL	+	+	+	+	+	+	+	+	+	+
K02711	PsbJ	+	+	+	+	+	+	+	+	+	+
K02712	PsbK	+	+	+	+	+	+	+	+	+	+
K02714	PsbM	+	+	+	+	+	+	+	+	+	+
K02709	PsbH	+	+	+	+	+	+	+	+	+	+
K02710	PsbI	+	+	+	+	+	+	-	+	-	-
K02716	PsbO	+	+	+	+	+	+	+	+	+	+
K02717	PsbP	+	+	+	+	+	+	+	+	+	+
K08901	PsbQ	-	-	-	-	-	-	-	-	-	-
K03541	PsbR	-	-	-	-	-	-	-	-	-	-
K03542	PsbS	-	-	-	-	-	-	-	-	-	-
K02718	PsbT	+	+	+	+	+	+	-	+	+	+
K02719	PsbU	+	+	+	+	+	+	+	+	+	+
K02720	PsbV	+	+	+	+	+	+	+	+	+	+
K02721	PsbW	-	-	-	-	-	-	-	-	-	-
K02722	PsbX	+	+	+	+	+	+	+	+	+	+
K02723	PsbY	+	+	+	+	+	+	+	+	+	+
K02724	PsbZ	+	+	+	+	++	+	+	+	+	+
K08902	Psb27	+	+	+	+	+	+	+	+	+	+
K08903	Psb28	+	+	+	+	+	+	+	+	+	+
K08904	Psb28-2	-	-	-	-	-	+	+	+	+	+
PSI											
K02689	PsaA	+	+	+	+	+	+	+	+	+	+
K02690	PsaB	+	+	+	+	+	+	+	+	+	+
K02691	PsaC	+	+	+	+	+	+	+	+	+	+
K02692	PsaD	+	+	+	+	+	+	+	+	+	+
K02693	PsaE	+	+	+	+	+	+	+	+	+	+
K02694	PsaF	+	+	+	+	+	+	+	+	+	+
K08905	PsaG	-	-	-	-	-	-	-	-	-	-
K02695	PsaH	-	-	-	-	-	-	-	-	-	-
K02696	PsaI	++	++	++	++	++	+	+	+	+	+
K02697	PsaJ	+	+	+	+	+	+	+	+	+	+
K02698	PsaK	+	+	+	+	+	++	++	++	++	++
K02699	PsaL	+	+	+	+	+	+	+	+	+	+
K02700	PsaM	+	+	+	+	+	+	+	+	+	+
K02701	PsaN	-	-	-	-	-	-	-	-	-	-
K14332	PsaO	-	-	-	-	-	-	-	-	-	-
K02702	PsaX	-	-	-	-	-	-	-	-	-	-
Cytochrome b6/f complex											
K02635	PetB	+	+	+	+	+	+	+	+	+	+
K02637	PetD	+	+	+	+	+	+	+	+	+	+
K02634	PetA	+	+	+	+	+	+	+	+	+	+
K02636	PetC	+	+	+	++	+	+	+	+	+	+
K02642	PetL	-	-	-	-	-	+	+	+	+	+
K02643	PetM	+	+	+	+	+	+	+	+	+	+
K03689	PetN	+	+	+	+	+	+	-	+	+	+
K02640	PetG	+	+	+	+	+	+	+	-	+	+
Photosynthetic electron transport											
K02638	PetE	-	-	-	-	-	+	+	+	+	+
K02639	PetF	++++	++++	++++	++++	++++	+++	+++	+++	+++	+++
K02641	PetH	+	+	+	+	+	+	+	+	+	+
K08906	PetJ	+	+	+	++	++	+++	+++	+++	+++	+++
F-type ATPase											
K02112	beta	+	+	+	+	+	+	+	+	+	+
K02111	alpha	+	+	+	+	+	+	+	+	+	+
K02115	gamma	+	+	+	+	+	+	+	+	+	+
K02113	delta	+	+	+	+	+	+	+	+	+	+
K02114	epsilon	+	+	+	+	+	+	+	+	+	+
K02110	c	+	+	+	+	+	+	+	+	+	+
K02108	a	+	+	+	+	+	+	+	+	+	+
K02109	b	++	++	++	++	++	++	++	++	++	++
Photosynthesis - Antenna Proteins											
Allophycocyanin (AP)											
K02092	ApcA	+	+	+	+	+	+	+	+	+	+
K02093	ApcB	+	+	+	+	+	+	+	+	+	+
K02094	ApcC	+	+	+	+	+	+	+	+	+	+
K02095	ApcD	+	+	+	+	+	++	++	+	++	++
K02096	ApcE	+	+	+	+	+	+	+	+	+	+
K02097	ApcF	+	+	+	+	+	+	+	+	+	+
Phycocyanin (PC)/Phycoerythrocyanin (PEC)											
K02284	CpcA	++	++	++	+	-	++	++	++	++	++
K02285	CpcB	+++	+++	+++	++	+	++	++	++	++	++
K02286	CpcC	-	-	-	-	-	++	++	++	++	++
K02287	CpcD	+	+	+	+	+	+	+	+	+	+
K02288	CpcE	+	+	+	+	+	+	+	+	+	+
K02289	CpcF	+	+	+	+	+	+	+	+	+	+
K02290	CpcG	++	++	++	++	++	+	+	+	+	+
Phycoerythrin (PE)											
K05376	CpeA	-	-	-	-	-	-	-	-	-	-
K05377	CpeB	-	-	-	-	-	-	-	-	-	-
K05378	CpeC	++	++	++	++	++	-	-	-	-	-
K05379	CpeD	-	-	-	-	-	-	-	-	-	-
K05380	CpeE	-	-	-	-	-	-	-	-	-	-
K05381	CpeR	-	-	-	-	-	-	-	-	-	-
K05382	CpeS	-	-	-	-	-	+	+	+	+	+
K05383	CpeT	-	-	-	-	-	-	-	-	-	-
K05384	CpeU	-	-	-	-	-	-	-	-	-	-
K05385	CpeY	-	-	-	-	-	-	-	-	-	-
K05386	CpeZ	-	-	-	-	-	-	-	-	-	-

## Abbreviations

AP: allophycocyanin
CCGs: core cyanobacterial genes
PBS: phycobilisome
PE: phycoerythrin
PSI: Photosystem I
PSII: Photosystem II
