# Supplementary material for: Draft Genome Sequences of Synechococcus sp. strains CCAP1479/9, CCAP1479/10, CCAP1479/13, CCY0621, and CCY9618: Five Freshwater Syn/Pro Clade Picocyanobacteria
Source: J Genomics. 2023 Apr 25;11:26–36. doi: 10.7150/jgen.81013 (PMC10161378; doi:10.7150/jgen.81013)
Supplement: Supplementary file 3 — Supplementary figure 2. [file jgenv11p0026s3.pdf]

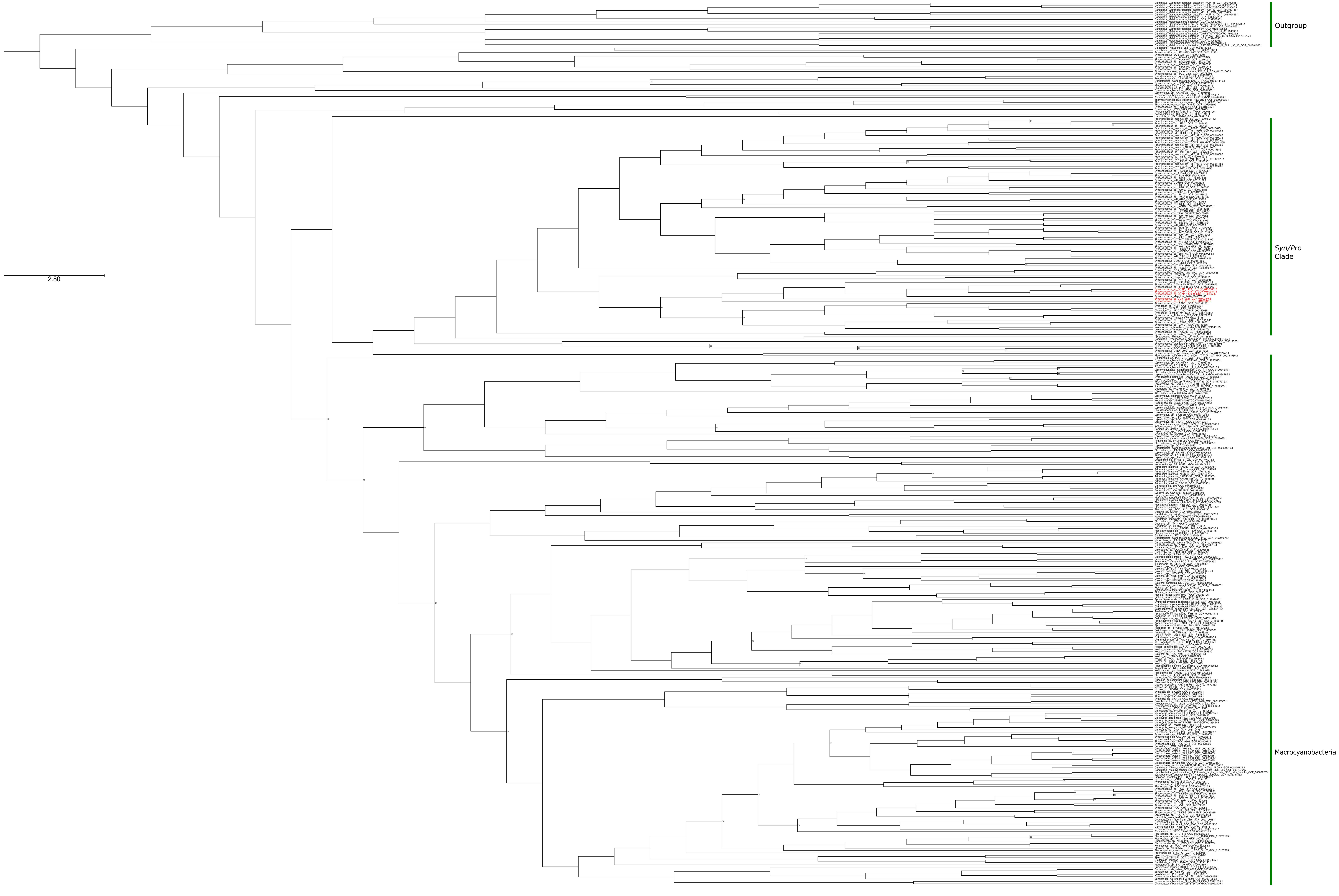

Outgroup
Syn/Pro Clade
Macrocyanobacteria
2.80
