# Supplementary material for: Draft Genome Sequences of Synechococcus sp. strains CCAP1479/9, CCAP1479/10, CCAP1479/13, CCY0621, and CCY9618: Five Freshwater Syn/Pro Clade Picocyanobacteria
Source: J Genomics. 2023 Apr 25;11:26–36. doi: 10.7150/jgen.81013 (PMC10161378; doi:10.7150/jgen.81013)

Supplementary Information:

Draft Genome Sequences of *Synechococcus* sp. strains CCAP1479/9, CCAP1479/10, CCAP1479/13, CCY0621, and CCY9618: Five Freshwater *Syn/Pro* Clade Picocyanobacteria

Elliot Druce[1], Michele Grego[2], Henk Bolhuis[3], Penny J. Johnes[1], Patricia Sánchez-Baracaldo[1]

[1] School of Geographical Sciences, Faculty of Science, University of Bristol, Bristol, BS8 1SS, United Kingdom.

[2] CNRS and Sorbonne Université, FR 2424, Roscoff Culture Collection, Station Biologique de Roscoff (SBR), Roscoff, France

[3] Department of Marine Microbiology and Biogeochemistry, Royal Netherlands Institute for Sea Research, Den Hoorn, the Netherlands.

Corresponding author

Name: Patricia Sánchez-Baracaldo

E-mail: p.sanchez-baracaldo@bristol.ac.uk

Includes:

Supplementary Figures S1 to S7

UOB Open

Supplementary Information

Figure S1: **Geographical location of picocyanobacterial strains**. *Synechococcus* sp. CCAP1479/9 and *Synechococcus* sp. CCAP1479/10 were both collected from the south basin of Lake Windermere (purple marker). *Synechococcus* sp. CCAP 1479/13 was collected from the north basin of Lake Windermere (blue marker). *Synechococcus* sp. CCY 0621 was isolated in Leiden, The Netherlands (green marker) and *Synechococcus* sp. CCY 9618 was isolated in Vinkeveen, The Netherlands (red marker).

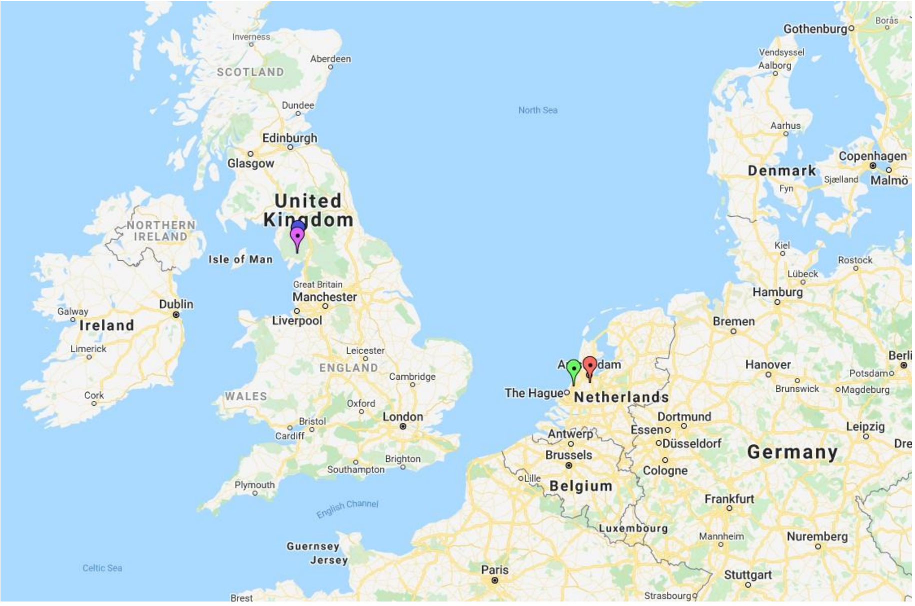

Figure S2: Expanded view of the phylogeny featuring all cyanobacteria utilised in a maximum likelihood analysis based off 145 orthologous proteins. Newly sequenced strains are highlighted in red. Bootstrap values less than 100 are displayed at nodes. The scale bar represents an average of 2.8 substitutions per site. For a full list of taxa included see Supplementary Table S4.

See attached file

Figure S3: Assembled reads of *Synechococcus* sp. CCY 0621 using De Bruijn visualisation. Output from Bandage v0.8.1. Nodes with a depth > 243 are in green, nodes with a depth < 15 are in lilac.

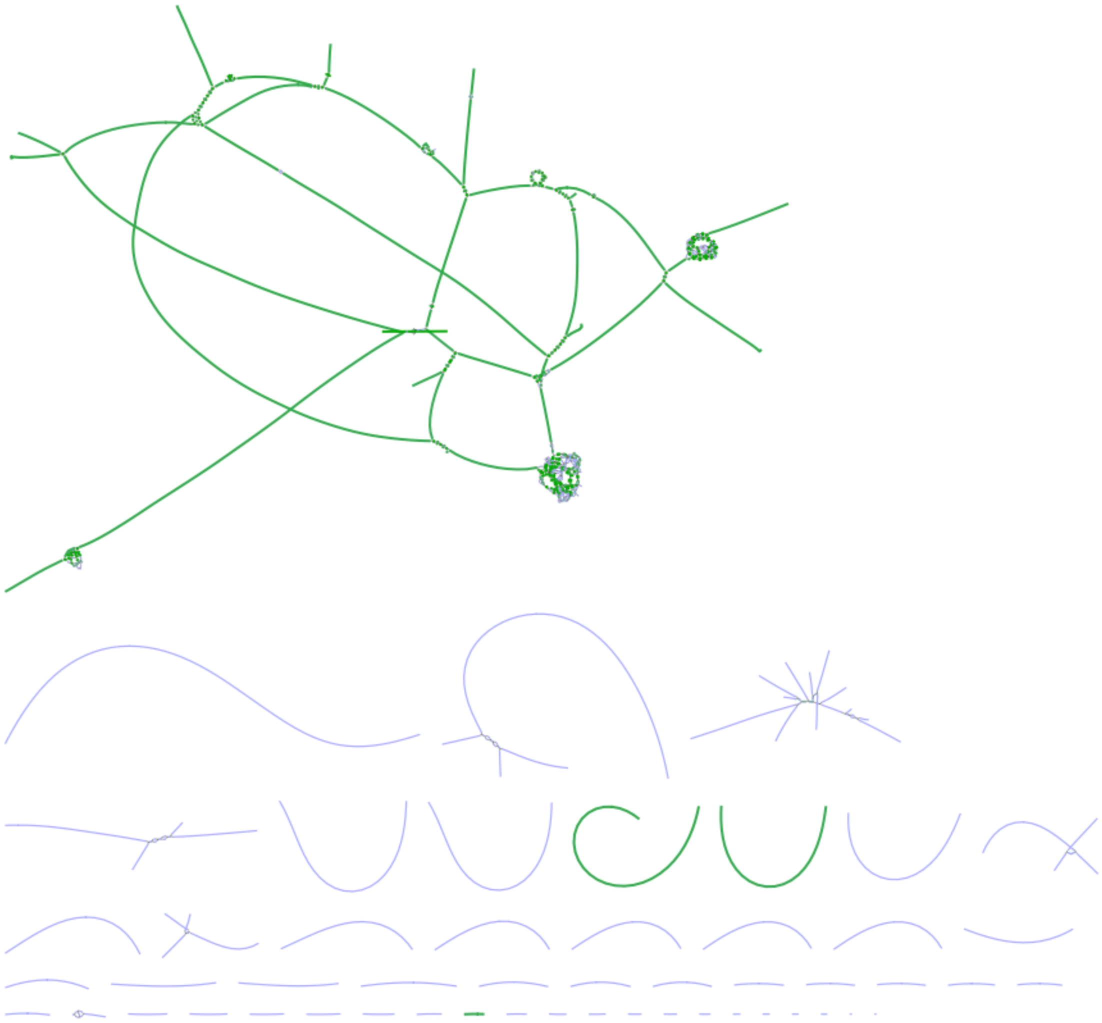

Figure S4: Assembled reads of *Synechococcus* sp. CCY 9618 using De Bruijn visualisation. Output from Bandage v0.8.1. Nodes with a depth > 175 are in green, nodes with a depth < 30 are in lilac.

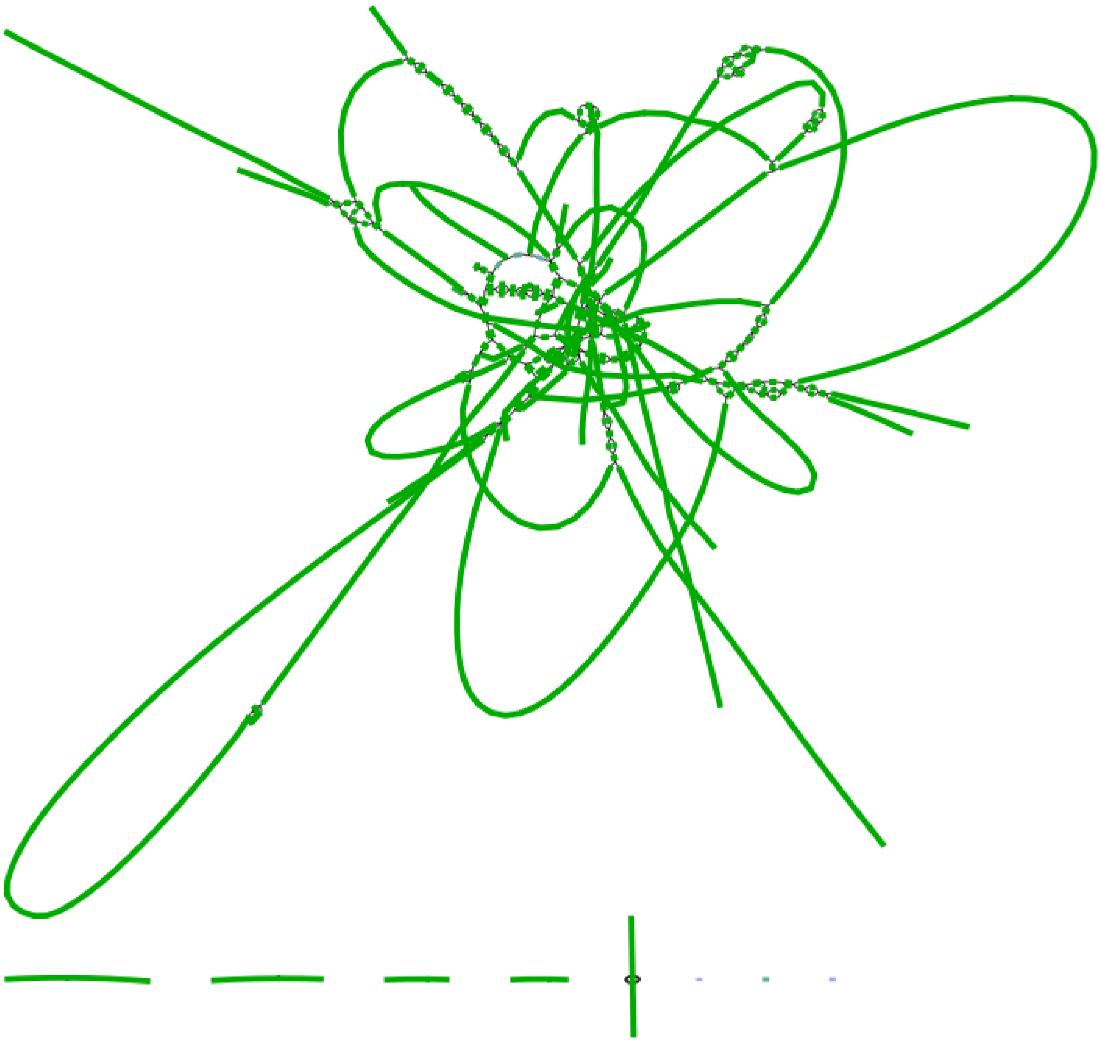

Figure S5: Assembled reads of *Synechococcus* sp. CCAP 1479/9 using De Bruijn visualisation. Output from Bandage v0.8.1. Nodes with a depth > 181 are in green, nodes with a depth < 21 are in lilac.

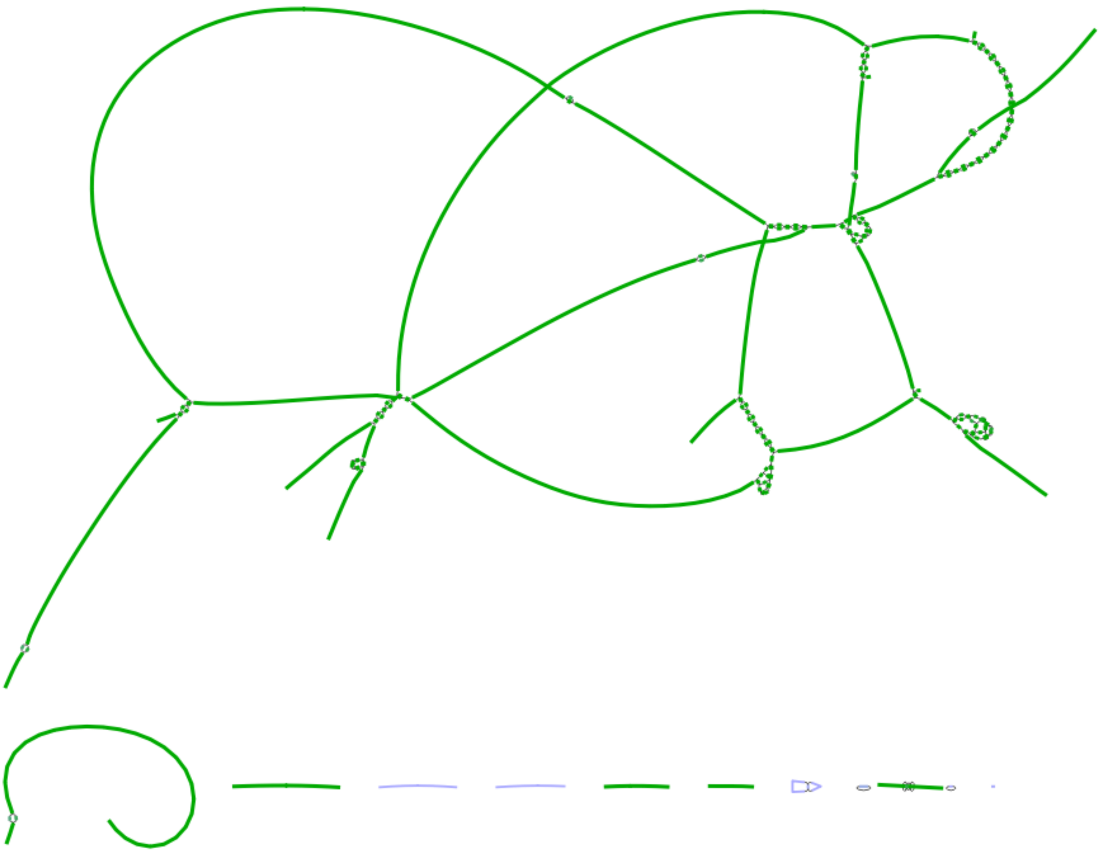

Figure S6: Assembled reads of *Synechococcus* sp. CCAP 1479/10 using De Bruijn visualisation. Output from Bandage v0.8.1. Nodes with a depth > 119 are in green, nodes with a depth < 17 are in lilac.

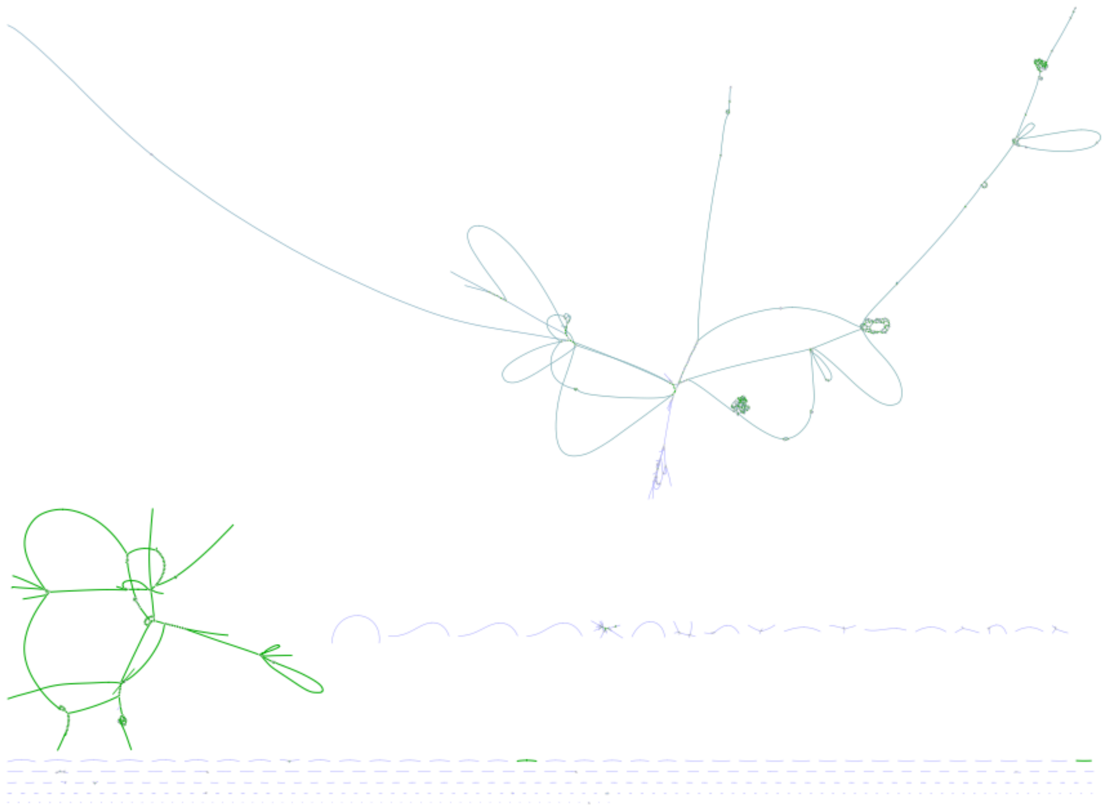

Figure S7: Assembled reads of *Synechococcus* sp. CCAP 1479/13 using De Bruijn visualisation. Output from Bandage v0.8.1. Nodes with a depth > 146 are in green, nodes with a depth < 27 are in lilac.

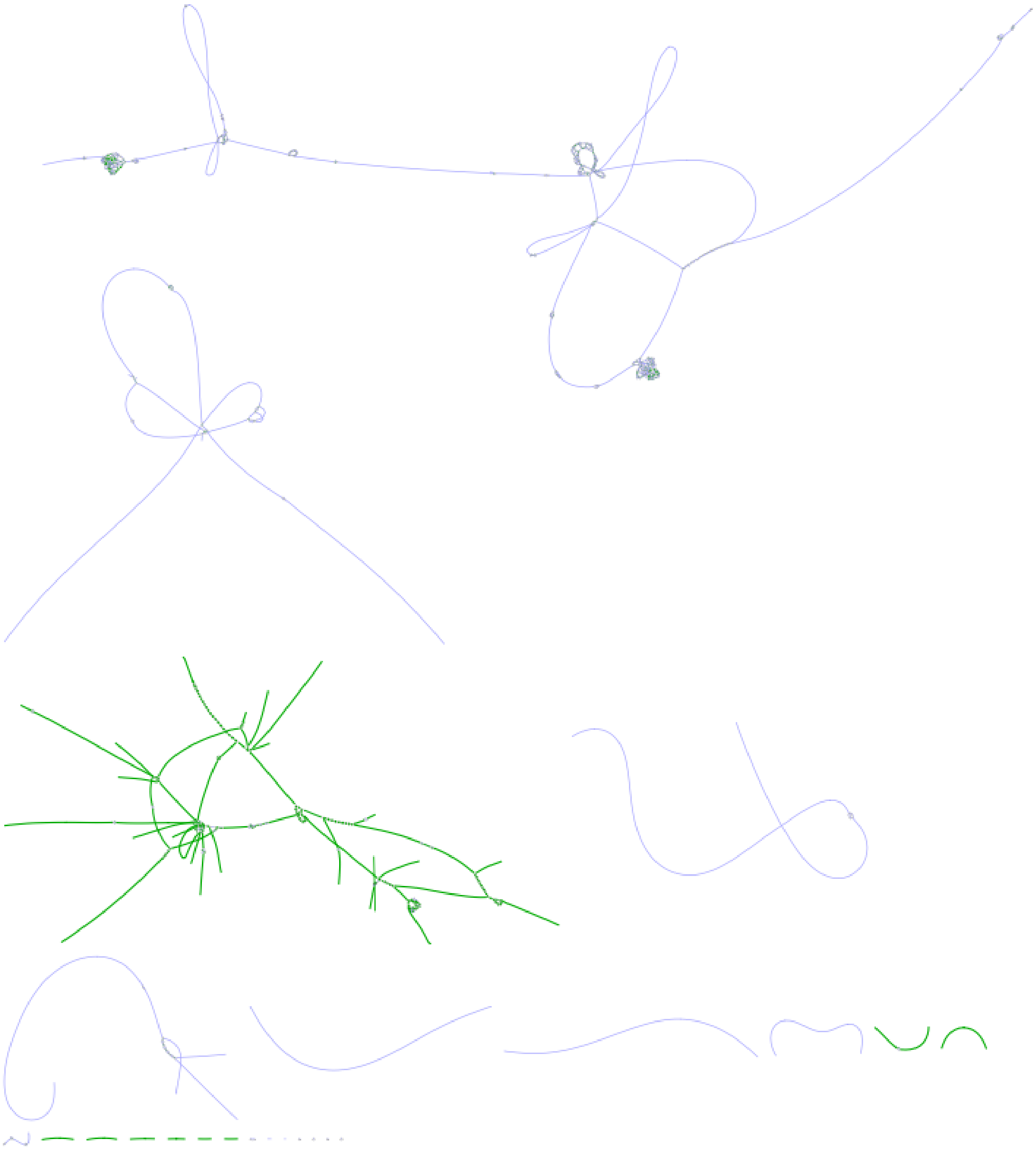

Supplement: Supplementary file 2 — Supplementary figures 1, 3-7. [file jgenv11p0026s2.pdf]
